# Melanoma-Targeted Chemothermotherapy and *In Situ* Peptide Immunotherapy through HSP Production by Using Melanogenesis Substrate, NPrCAP, and Magnetite Nanoparticles

**DOI:** 10.1155/2013/742925

**Published:** 2013-02-21

**Authors:** Kowichi Jimbow, Yasue Ishii-Osai, Shosuke Ito, Yasuaki Tamura, Akira Ito, Akihiro Yoneta, Takafumi Kamiya, Toshiharu Yamashita, Hiroyuki Honda, Kazumasa Wakamatsu, Katsutoshi Murase, Satoshi Nohara, Eiichi Nakayama, Takeo Hasegawa, Itsuo Yamamoto, Takeshi Kobayashi

**Affiliations:** ^1^Institute of Dermatology & Cutaneous Sciences, 1-27 Odori West 17, Chuo-ku, Sapporo 060-0042, Japan; ^2^Department of Dermatology, School of Medicine, Sapporo Medical University, South 1 West 16, Chuo-ku, Sapporo 060-8556, Japan; ^3^Department of Chemistry, School of Health Sciences, Fujita Health University, 1-98 Dengakugakubo, Kutsukake-cho, Toyoake, Aichi 470-1192, Japan; ^4^Department of Pathology 1, School of Medicine, Sapporo Medical University, South 1 West 16, Chuo-ku, Sapporo 060-8556, Japan; ^5^Department of Chemical Engineering, Faculty of Engineering, Kyushu University, 744 Motooka, Nishi-ku, Fukuoka 819-0395, Japan; ^6^Department of Biotechnology, School of Engineering, Nagoya University, Furo-cho, Chikusa-ku, Nagoya 464-8603, Japan; ^7^Meito Sangyo Co., Ltd., 25-5 Kaechi, Nishibiwajima-cho, Kiyosu, Aichi 452-0067, Japan; ^8^Faculty of Health and Welfare, Kawasaki University of Medical Welfare, 288 Matsushimai, Kurashiki, Okayama 701-0193, Japan; ^9^Department of Hyperthermia Medical Research Laboratory, Louis Pasteur Center for Medical Research, 103-5, Tanakamonzen-cho, Sakyo-ku, Kyoto 606-8225, Japan; ^10^Yamamoto Vinita Co., Ltd., 3-12 ueshio 6, Tennoji-ku, Osaka 543-0002, Japan; ^11^Department of Biological Chemistry, College of Bioscience and Biotechnology, Chubu University, 1200 Matsumoto-cho, Kasugai, Aichi 487-8501, Japan

## Abstract

Exploitation of biological properties unique to cancer cells may provide a novel approach to overcome difficult challenges to the treatment of advanced melanoma. In order to develop melanoma-targeted chemothermoimmunotherapy, a melanogenesis substrate, N-propionyl-4-S-cysteaminylphenol (NPrCAP), sulfur-amine analogue of tyrosine, was conjugated with magnetite nanoparticles. NPrCAP was exploited from melanogenesis substrates, which are expected to be selectively incorporated into melanoma cells and produce highly reactive free radicals through reacting with tyrosinase, resulting in chemotherapeutic and immunotherapeutic effects by oxidative stress and apoptotic cell death. Magnetite nanoparticles were conjugated with NPrCAP to introduce thermotherapeutic and immunotherapeutic effects through nonapoptotic cell death and generation of heat shock protein (HSP) upon exposure to alternating magnetic field (AMF). During these therapeutic processes, NPrCAP was also expected to provide melanoma-targeted drug delivery system.

## 1. Introduction

The incidence of melanoma is increasing worldwide at an alarming rate [1, 2]. As yet, management of metastatic melanoma is an extremely difficult challenge. Less than 10% with metastatic melanoma patients survive currently for five years because of the lack of effective therapies [3]. There is, therefore, an emerging need to develop innovative therapies for the control of metastatic melanoma.

**Figure 1:**
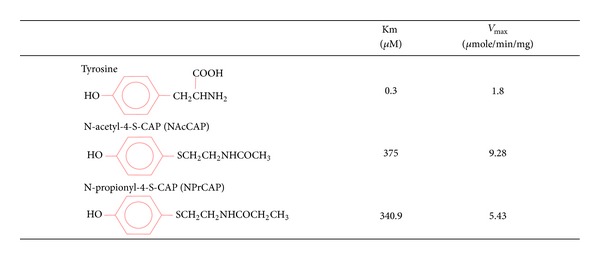
Synthesis and chemical structures of NAcCAP and NPrCAP and their tyrosinase kinetics.

The major advance of drug discovery for targeted therapy to cancer cells can be achieved by exploiting their unique biological property. The biological property unique to the melanoma cell resides in the biosynthesis of melanin pigments, that is, melanogenesis occuring within specific compartments, melanosomes. Melanogenesis begins with the conversion of amino acid, tyrosine to dopa and subsequently to dopaquinone in the presence of tyrosinase. This pathway is uniquely expressed by all melanoma cells. It is well known that the clinically “amelanotic” melanoma tissues always have tyrosinase activity to some extent, and that “*in vitro* amelanotic” melanoma cells become “melanotic” ones when they are regrown in the *in vivo* condition. Melanin precursors are inherently cytotoxic through reacting with tyrosinase to form unstable quinone derivatives [4]. Thus, tyrosine analogues that are tyrosinase substrates can be good candidates for developing drugs to melanoma-targeting therapies [5]. N-propionyl and N-acetyl derivatives (NPr- and NAcCAP) of 4-*S*-cysteaminylphenol, that is, sulfur-amine analogue of tyrosine, were synthesized as possible melanoma-targeted drugs (Figure 1) and found to possess selective cytotoxic effects on *in vivo* and *in vitro* melanomas through the oxidative stress that derives from production of cytotoxic free radicals by interacting with tyrosinase within melanogenesis cascade [6–10].

Intracellular hyperthermia using magnetite nanoparticles (10–100 nm-sized Fe_3_O_4_) may be another choice to overcome the difficult challenges for melanoma treatment. It has been shown to be effective for treating cancers in not only primary but also metastatic lesions [11, 12]. Incorporated magnetite nanoparticles generate heat (thermotherapy) within the cells after exposure to AMF due to hysteresis loss [13]. In this treatment, there is not only the heat-mediated cell death but also immune reaction due to the generation of heat shock proteins (HSPs) [14–23]. HSP expression induced by hyperthermia has been shown to be involved in tumor immunity, providing the basis for developing a cancer thermoimmunotherapy.

**Figure 2:**
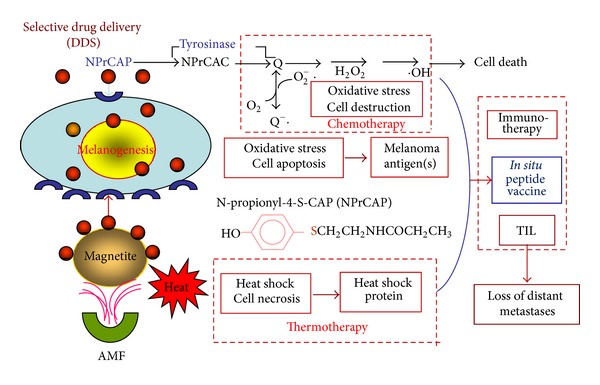
Strategy for melanogenesis-targeted CTI and *in situ* peptide vaccine therapy by conjugates of NPrCAP and magnetite nanoparticles with AMF exposure.

Based upon these rationales, we now provide evidence that melanoma-targeted chemothermotherapy can be achieved by conjugating a chemically modified melanogenesis substrate, NPrCAP with magnetite nanoparticles, which then produce apoptotic and non-apoptotic cell death through interacting with tyrosinase and heat-mediated oxidative stress; hence, immunotherapy with production of* in situ *peptides is being established (Figure 2).

## 2. Melanogenesis Substrate as a Potential Candidate for Development of Selective Drug Delivery System and Cytotoxicity to Melanoma

### 2.1. Synthesis of Sulfur-Amine Analogues of Tyrosine, Cysteaminylphenols, and Their Selective Incorporation into Melanogenesis Cascade

With the interaction of melanocyte-stimulating hormone (MSH)/melanocortin 1 receptor (MC1R), the melanogenesis cascade begins from activation of microphthalmia transcription factor (MITF) for induction of either eu- or pheomelanin biosynthesis. Tyrosinase is the major player in this cascade. Tyrosinase is a glycoprotein, and its glycosylation process is regulated by a number of molecular chaperons, including calnexin in the endoplasmic reticulum [24, 25]. Vesicular transport then occurs to carry tyrosinase and its related proteins (TRPs) from trans-Golgi network to melanosomal compartments, which appear to derive from early and late endosomal compartments. In this process a number of transporters, such as small GTP-binding protein, adaptor proteins, and PI3-kinase, play important roles. Once melanin biosynthesis is completed to conduct either eu- or pheomelanogenesis within melanosomes, they then move along dendritic processes and are transferred to surrounding keratinocytes in normal skin [26–28]. In metastatic melanoma cells, however, there will be practically no melanosome transfer inasmuch as there will be no receptor cells such as keratinocytes. Thus melanosomes synthesized by melanoma cells are aggregated within autophagic vacuoles in which melanogenesis-targeted drugs will be retained. In order to utilize this unique melanogenesis pathway for developing melanoma-targeted drugs, N-acetyl and N-propionyl derivatives of cysteaminylphenols (NAc- and NPrCAPs) have been synthesized [8, 29] (Figure 1).

### 2.2. *In Vivo* and *In Vitro* Melanocyte Toxicity and Anti-Melanoma Effects of Cysteaminylphenols (CAPs)

**Figure 3:**
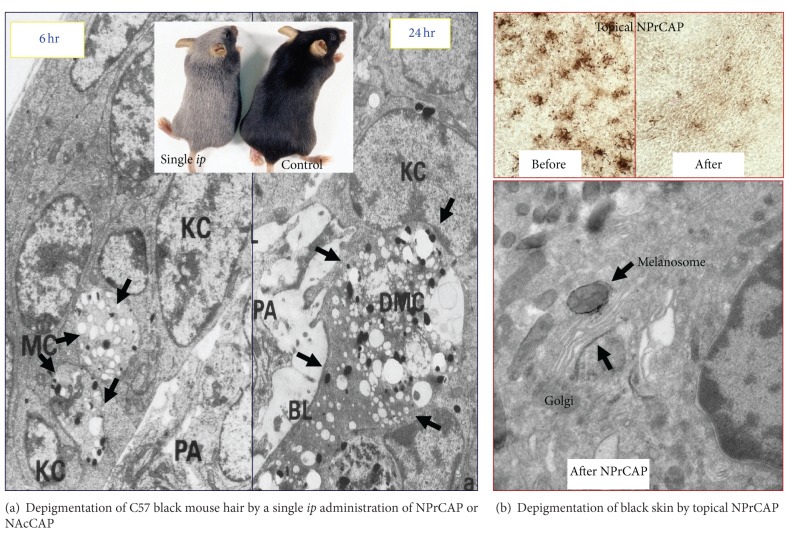
Depigmenting effect of NPrCAP. (a) Depigmentation of C57 black mouse hair follicles by a single *ip* administration of NPrCAP or NAcCAP results in complete loss of melanin pigmentation. Entire coat color changes to silver from black. Electron microscopic observation reveals selective degradation of melanocytes and melanogenic organelles such as early-stage melanosomes at 6 hr after administration. At 24 hr after administration, these melanocytes reveal total degradation. (b) Depigmentation of black skin after topical application of NPrCAP. There is a marked decrease of melanocyte populations after topical application. Electron microscopic observation indicates selective accumulation of NPrCAP in the tyrosinase areas such as in melanosomes and Golgi apparatus as indicated by the deposition of electron dense materials (see arrows).

Both NPrCAP and NAcCAP were found to selectively disintegrate follicular melanocytes after single or multiple* ip* administration to newborn or adult C57 black mice, respectively [12, 30]. In the case of adult mice after repeated *ip* administration of NPrCAP, white follicles with 100% success rate can be seen at the site where hair follicles were plucked to stimulate new melanocyte growth and to activate new tyrosinase synthesis. A single *ip* administration of NPrCAP into a new born mouse resulted in the development of silver follicles in the entire body coat. The selective disintegration of melanocytes which is mediated by apoptotic cell death can be seen as early as in 12 hr after a single *ip* administration. None of surrounding keratinocytes or fibroblasts showed such membrane degeneration and cell death [31, 32] (Figure 3).

A high, specific uptake of NAcCAP was seen* in vitro* by melanoma cell lines compared to nonmelanoma cells [9]. A melanoma-bearing mouse showed, on the whole body autoradiogram, the selective uptake and covalent binding of NAcCAP in melanoma tissues of lung and skin [6]. The specific cytotoxicity of NPrCAP and NAcCAP was examined on various types of culture cells by MTT assay, showing that only melanocytic cells except HeLa cells possessed the low IC50 [8, 9]. The cytotoxicity on DNA synthesis inhibition was timedependent and irreversible on melanoma cells but was transient on HeLa cells [10].

The *in vitro* culture and *in vivo* lung metastasis assays showed the melanoma growth can be blocked by administration of NAcCAP combined with buthionine sulfoximide (BSO), which blocked the effect of antioxidants through reducing glutathione levels. There was a marked growth inhibition of cultured melanoma cells in the presence of BSO indicating that the selective cytotoxicity by CAP is mediated by the production of cytotoxic free radicals. The *in vivo* lung metastasis experiment also showed the decreased number of lung melanoma colonies [6]. The problem was, however, that a fairly large number of amelanotic melanoma lesions were seen to grow in the lung [6]. NPrCAP has been developed and conjugated with magnetite nanoparticles in the hope of increasing the cytotoxicity and overcoming the problem.

## 3. Conjugation of NPrCAP with Magnetite Nanoparticles and *In Vivo* Evaluation of Melanoma Growth Inhibition with/without Thermotherapy

### 3.1. Synthesis for Conjugates of NPrCAP with Magnetite Nanoparticles and Their Selective Aggregation in Melanoma for Development of Chemo-Thermo-Immunotherapy

Magnetite nanoparticles have been employed for thermotherapy in a number of cancer treatments including human gliomas and prostate cancers [33–35]. They consist of 10–100 nm-sized iron oxide (Fe_3_O_4_) with a surrounding polymer coating and generate heat when exposed to AMF [12]. We expected the combination of NPrCAP and magnetite nanoparticles to be a potential source for developing not only antimelanoma pharmacologic but also immunogenic agent. Based upon the melanogenesis-targeted drug delivery system (DDS) of NPrCAP, NPrCAP/magnetite nanoparticles complex was expected to be selectively incorporated into melanoma cells. It was also hypothesized that the degradation of melanoma tissues may occur from oxidative and heat stresses by exposure of NPrCAP to tyrosinase and by exposure of magnetite nanoparticles to AMF. These two stress processes may then produce the synergistic or additive effect for generating tumor-infiltrating lymphocytes (TIL) by *in situ* formation of peptides that will kill melanoma cells in distant metastases (Figure 2). 

**Figure 4:**
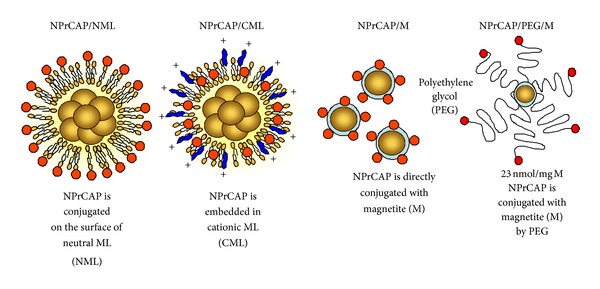
Conjugates of NPrCAP/magnetite nanoparticles for developing melanogenesis-targeted melanoma nanomedicine.

In order to develop effective melanoma-targeted chemotherapy (by NPrCAP) and thermo-immunotherapy (by magnetite nanoparticles with HSP), hence providing a basis for chemo-thermo-immunotherapy (CTI therapy), we synthesized conjugates of NPrCAP and magnetite nanoparticles, on which NPrCAP is bound directly or indirectly on the surface of magnetite nanoparticles or magnetite-containing liposomes (Figure 4). Among these NPrCAP and magnetite complexes listed in Figure 4, NPrCAP/M and NPrCAP/PEG/M were chemically stable, did not lose biological property, and could be filtered as well as easily produced in large quantities. Most of the experiments described below were carried out by employing the direct conjugate of NPrCAP and magnetite nanoparticles, NPrCAP/M. A preliminary clinical trial, however, used NPrCAP/PEG/M to which polyethylene glycol (PEG) was employed to conjugate NPrCAP and magnetite nanoparticles.

In our studies, we found that NPrCAP/M nanoparticle conjugates were selectively aggregated in melanoma cells compared to non-melanoma cells [36]. The conjugates of NPrCAP and magnetite nanoparticles would be selectively aggregated on the cell surface of melanoma cells through still unknown surface receptor and then incorporated into melanoma cells by early and late endosomes. The conjugates were then incorporated into melanosomal compartment as the stage I melanosomes derive from late endosome-related organelles, to which tyrosinase was transported from the trans-Golgi network by vesicular transport [26].

### 3.2. *In Vivo *Growth Inhibition of Mouse Melanoma by Conjugates of NPrCAP and Magnetite Nanoparticles with/without Thermotherapy

The intracellular hyperthermia using magnetic nanoparticles is effective for treating certain types of primary and metastatic cancers [11, 12, 35–39]. Incorporated magnetic nanoparticles generate heat within the cells after exposure to the AMF due to hysteresis loss or relaxational loss [13, 40]. In our study of B16 melanoma cells using B16F1, B16F10, and B16OVA cells, we compared the thermo-therapeutic protocols in detail by evaluating the growth of the rechallenge melanoma transplants as well as the duration and rates of survival of melanoma-bearing mice. 

**Figure 5:**
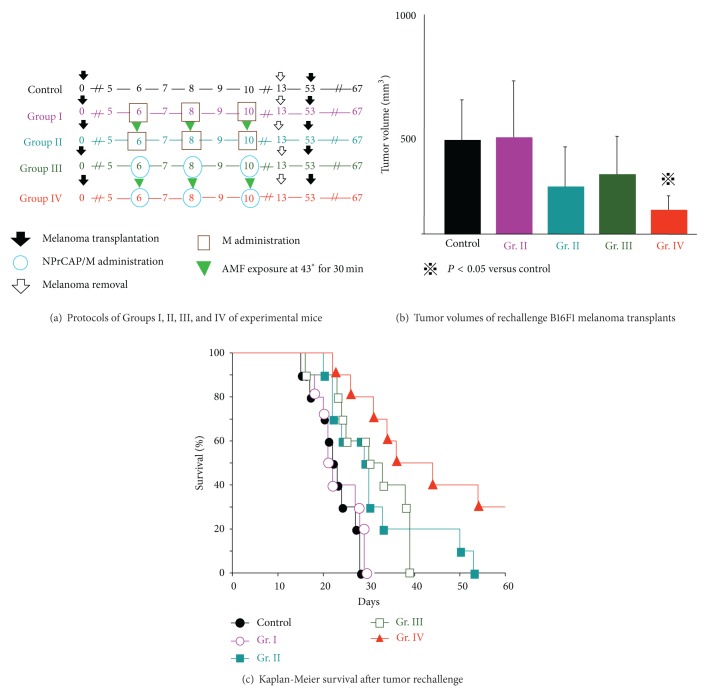
Melanoma growth and survival of melanoma-bearing mice by CTI therapy using NPrCAP/M with and without AMF exposure. (a) Experimental protocols. (b) Tumor volumes of rechallenge melanoma transplants on day 13 of after transplantation. (c) Kaplan-Meier survival of melanoma-bearing mice after treatment following experimental protocols of Figure 5(a).

By employing B16F1 and F10 cells, we first evaluated the chemotherapeutic effect of NPrCAP/M with or without AMF exposure which generates heat. NPrCAP/M without heat inhibited growth of primary transplants to the same degree as did NPrCAP/M with heat, indicating that NPrCAP/M alone has a chemotherapeutic effect. However, there was a significant difference in the melanoma growth inhibition of re-challenge transplants between the groups of NPrCAP/M with and without heat. NPrCAP/M with AMF exposure showed the most significant growth inhibition in re-challenge melanoma transplants and increased life span of the host animals, that is, almost complete rejection of re-challenge melanoma growth, whereas NPrCAP/M without heat was much less, indicating that NPrCAP/M with heat possesses a thermo-immunotherapeutic effect (Figures 5(a), 5(b), and 5(c)).

Specifically our study indicated that the most effective thermoimmunotherapy for re-challenge B16F1 and F10 melanoma cells can be obtained at a temperature of 43°C for 30 min with the treatment repeated three times on every other day intervals without complete degradation of the primary melanoma [37]. This therapeutic approach and its biologic effects differ from those of magnetically mediated hyperthermia on the transplanted melanomas reported previously. In previous studies by Suzuki et al. [38]and Yanase et al. [39], cationic magnetoliposomes were used for B16 melanoma. They showed that hyperthermia at 46°C once or twice led to regression of 40–90% of primary tumors and to 30–60% survival of mice, whereas their hyperthermia at 43°C failed to induce regression of the secondary tumors and any increase of survival in mice [38, 39].

## 4. Production of Heat Shock Protein, Nonapoptotic Cell Death, and Tumor-Infiltrating Lymphocytes by Conjugates of NPrCAP and Magnetite Nanoparticles with Thermotherapy

### 4.1. Production of Heat Shock Protein and Non-Apoptotic Melanoma Cell Death by NPrCAP/Magnetite Nanoparticle Conjugates with Thermotherapy

**Figure 6:**
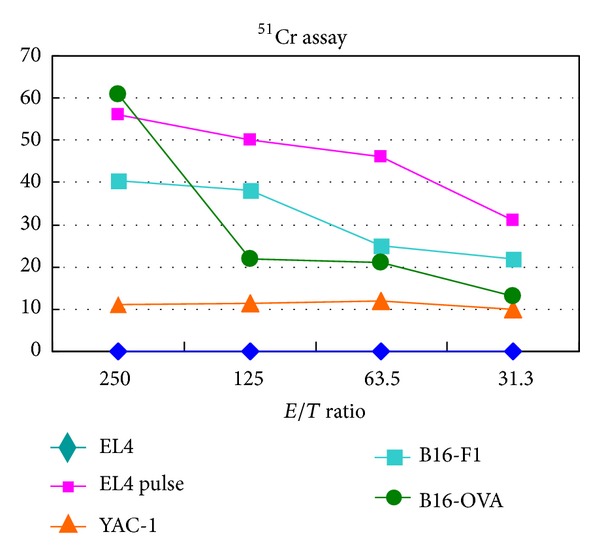
Hyperthermia of melanoma cells using B16OVA cells for induction of CTL in CTI therapy. Cytotoxic activity of spleen cells after CTI therapy against B16OVA cells, B16F1 cells, EL4 cells, EL4 cells pulsed with SL8 peptide (OVA-immunodominant peptide), or YAC-1 cells was determined by standard ^51^Cr-release assay. B16OVA cells were subjected to hyperthermia using NPrCAP/M with AMF exposure *in vitro*.

It has been shown that hyperthermia treatment using magnetite cationic liposomes (MCLs), which are cationic liposomes containing 10-nm magnetite nanoparticles, induced antitumor immunity through HSP expression [12, 22, 41, 42]. In our studies using B16F1, F10, and OVA melanoma cells [43], the hyperthermia using NPrCAP/M with AMF exposure also showed antitumor immune responses via HSP-chaperoned antigen (Figure 6) [43]. It may be speculated that the HSPs-antigen peptide complex released from melanoma cells treated with this intracellular hyperthermia is taken up by dendritic cells (DCs) and cross-presented HSP-chaperoned peptide in the context of MHC class I molecules [44]. In our CTI therapy with AMF exposure, the heat-mediated melanoma cell necrosis was induced to NPrCAP/M-incorporated cells. In this group, we also found that repeated hyperthermia (3 cycles of NPrCAP/M administration and AMF irradiation) was required to induce the maximal antitumor immune response [37].

If melanoma cells escaped from this necrotic cell death, repeated hyperthermia should produce further necrotic cell death to the previously heat-shocked melanoma cells in which HSPs were induced. Our CTI therapy with AMF exposure using B16OVA cells showed that Hsp72/Hsc73, Hsp90, and ER-resident HSPs participated in the induction of CD8^+^ T-cell response [43]. Different from the results of B16F1 and F10 cells, Hsp72 was largely responsible for the augmented antigen presentation to CD8^+^ T cells. As Hsp72 is known to upregulate in response to hyperthermia or heat shock treatment [41], newly synthesized Hsp72 has a chance to bind to the heat-denatured melanoma-associated antigen.

### 4.2. T-Cell Receptor Repertoires of Tumor-Infiltrating Lymphocytes by Conjugates of NPrCAP and Magnetite Nanoparticles with Heat Exposure (Hyperthermia)

It is clear now from our previous studies [22, 41] that conjugates of NPrCAP/magnetite nanoparticles (NPrCAP/M) with heat treatment (hyperthermia) can successfully induce the growth inhibition of primary and secondary melanoma transplants. It is also found that NPrCAP/M with hyperthermia elicited the response of cytotoxic T lymphocyte (CTL) via the release of HSP-peptide complex from degraded tumor cells [43] (Figure 6). In addition, CD8^+^ T cells were observed within B16 melanoma nodules after hyperthermia using NPrCAP/M [37]. TIL reactivity to antigen is mediated via T-cell receptors (TCRs) consisting of *α* and *β* chains. We studied the TCR repertoire after hyperthermia using NPrCAP/M in order to further understand the T-cell response to melanoma after hyperthermia using NPrCAP/M [45]. We found that TCR repertoire was restricted in TILs, and the expansion of V *β* 11^+^ T cells was preferentially found. DNA sequences of the third complementarity determining regions were identified. This approach is based on subcutaneous melanoma transplantation in the hind foot pad, which confines the DLN to the inguinal and popliteal lymph nodes. Melanoma growth was significantly suppressed by the treatment of NPrCAP/M-mediated hyperthermia. CD8^+^ T cells were observed substantially around the tumor and slightly within the tumor, while few and no CD8^+^ T cells were observed around and within the tumor of nontreated mice. 

In addition, significant enlargement of inguinal DLNs was observed in all of tumor-bearing mice including non-treated mice and NPrCAP/M-injected mice. The number of CD8^+^ T cells in inguinal DLNs increased significantly in the mice treated with NPrCAP/M-mediated hyperthermia.

## 5. Melanocytotoxic and Immunogenic Properties of NPrCAP without Hyperthermia

### 5.1. Induction of Apoptosis, Reactive Oxygen Species (ROS), and Tumor-Specific Immune Response by NPrCAP Administration Alone

In our animal study, those animals bearing B16F1 and B16F10 melanoma cells showed, to certain degree, rejection of second re-challenge melanoma transplantation by administration of both NPrCAP alone and NPrCAP/M minus AMF exposure [46]. Our working hypothesis for this finding is that there is a difference in the cytotoxic mechanism and immunogenic property of NPrCAP/M between experimental groups with and without hyperthermia by AMF exposure. The animals with NPrCAP/M without AMF exposure resulted in non-necrotic, apoptotic cell death. The animals with NPrCAP/M plus AMF exposure, on the other hand, resulted in nonapoptotic, necrotic cell death with immune complex production of melanoma peptide as well as Hsp70 and a small amount of Hsp 90. 

**Figure 7:**
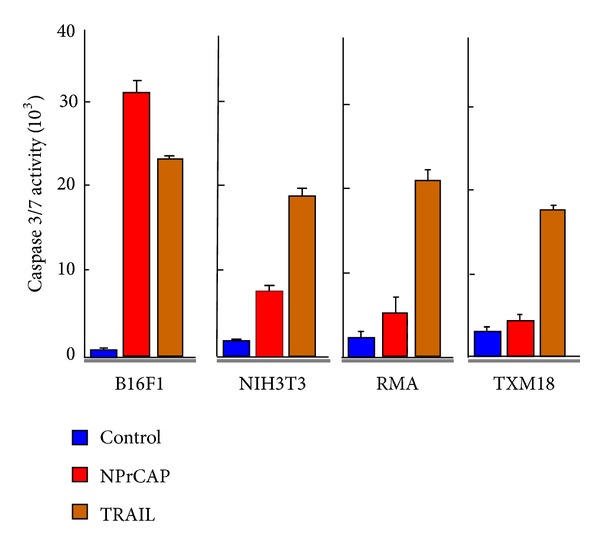
NPrCAP-mediated apoptotic cell death of B16F1 melanoma cells. Assay of caspase 3/7 in cells treated with NPrCAP or TRAIL. Cells were cultured in the presence of NPrCAP, TRAIL, or propylene glycol in 96-well plates and then processed for measurement of caspases 3 and 7 using a Caspase-Glo3/7 assay kit. From Ishii-Osai et al. [46].

To further examine the mechanism of the cell death induced by NPrCAP, those cells treated with NPrCAP alone were subjected to flow cytometric analysis, caspase 3 assay, and TUNEL staining [46]. The sub-G1 fraction was increased in the NPrCAP-treated B16F1 cells, comparable to TRAIL-exposed B16F1, but not in the NPrCAP-treated non-melanoma cells (NIH3T3, RMA) or nonpigmented melanoma cells (TXM18) (Figure 7). The luminescent assay detected caspase 3/7 activity in the NPrCAP-treated B16F1 cells remarkably increased (35.8-fold) compared to that in the nontreated cells. NIH3T3, RMA, and TXM18 cells treated with TRAIL showed 10.6-, 7.1-, and 5.8-fold increases of caspase 3/7 activation compared to the control, respectively, whereas those with NPr-4-*S*-CAP showed increases of 4.1-, 1.4-, and 1.8-fold, respectively. The number of TUNEL-positive cells was significantly increased only in the B16F1 tumor treated with NPrCAP. This increase was not observed in the B16F1 tumor without NPrCAP or in the RMA tumors with or without NPrCAP. The findings indicate that NPrCAP induces apoptotic cell death selectively in melanoma cells.

### 5.2. Melanocytotoxic and Immunogenic Properties of NPrCAP Compared to Monobenzyl Ether Hydroquinone

Monobenzyl ether of hydroquinone has long been known to produce the skin depigmentation at both the drug-applied area by direct chemical reaction with tyrosinase and the non-applied distant area by immune reaction with still unknown mechanism [43, 48–50]. The melanogenesis-related cytotoxicity primarily derives from tyrosinase-mediated formation of dopaquinone and other quinone intermediates, which produce ROSs such as superoxide and H_2_O_2_ [4, 31, 32, 51]. This unique biological property of melanin intermediates not only causes cell death, but also may produce immunogenic properties. We postulated that the cytotoxic action of NPrCAP appears to involve two major biological processes. One is cytostatic process which derives from the DNA synthesis inhibition through the interaction of quinone and free radicals with SH enzymes and thymidine synthase. Another is the cytocidal process by damage of DNA and mitochondrial ATP through oxidative stress and interaction with SH-enzyme [10]. They bind protein disulphide isomerase [52].

Monobenzyl ether form of hydroquinone was shown to produce a reactive *ortho*-quinone generated by tyrosinase-catalyzed oxidation and self-coupling and thiol conjugation reactions [53]. It was also shown to induce cell death without activating the caspase cascade or DNA fragmentation, indicating that the death pathway is non-apoptotic [53, 54]. It was further suggested that monobenzyl ether hydroquinone induced the immunogenicity to melanocytes and melanoma cells by forming quinone-haptens to tyrosinase protein and by inducing the release of tyrosinase and melanoma antigen recognized by T cells-1 (MART-1) containing CD63^+^ exosomes following melanosome oxidative stress induction. The drug further augmented the processing and shedding of melanocyte differentiation antigens by inducing melanosome autophagy and enhanced tyrosinase ubiquitination, ultimately activating dendritic cells, which induced cytotoxic melanoma-reactive cells. These T cells eradicated melanoma* in vivo *[54, 55].

### 5.3. Development of Vitiligo during Melanoma Immunotherapy and Activation of NPrCAP by Tyrosinase to Form Possible Antigen Peptides

Advanced melanoma patients and melanoma patients treated by vaccine immunotherapy often reveal vitiligo-like changes of the skin. Interestingly, this vitiligo development is associated with a superior prognosis in melanoma patients [56]. Although there have been several separate theories for the pathogenesis of vitiligo, the haptenation theory has recently been put forth to explain the molecular mechanism of monobenzone-induced skin depigmentation [54, 57, 58]. Westerhof et al. proposed the haptenation theory in which increased intracellular H_2_O_2_ could trigger the increased turnover of elevated levels of surrogate substrates of tyrosinase, resulting in melanocyte-specific T-cell responses [57, 59]. According to this hypothesis, tyrosinase could be recognized as a melanoma-specific tumor antigen in relation to the systemic immune responses. 

**Figure 8:**
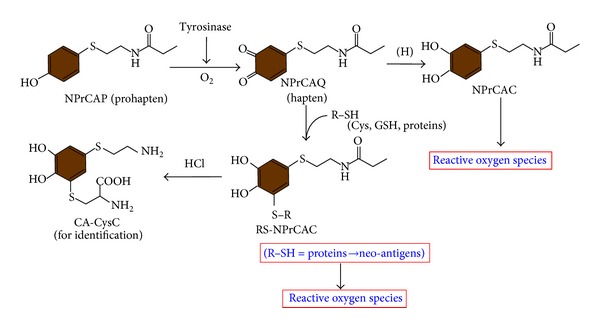
Tyrosinase activation of NPrCAP (prohapten) and binding of the quinone-hapten NPrCAQ with proteins thorough cysteine residues. Oxidation of NPrCAP with tyrosinase produces the quinone NPrCAQ, which is reduced to the catechol NPrCAC or binds to thiols (cysteine, glutathione, melanosomal proteins). The production of NPrCAQ-thiol adducts can be confirmed by the detection of CA-CysC after acid hydrolysis. NAcCys-NPrCAC is produced by the addition reaction of NAcCys (R-SH) with NPrCAQ. From Ito et al. [47].

Phenolic substrates as prohaptens are oxidized by tyrosinase to produce *ortho*-quinones, which act as haptens that covalently bind to tyrosinase or other melanosomal proteins to generate possible neoantigens [44, 53, 54]. These neo-antigens, in turn, trigger an immunological response cascade that results in a melanocyte-specific delayed-type hypersensitivity reaction leading to melanocyte elimination to produce depigmentation in vitiligo and melanoma rejection. We examined the tyrosinase-mediated oxidation of NPrCAP and its subsequent binding to sulfhydryl compounds (thiols) in NPrCAP-treated melanoma tissues and demonstrated that NPrCAP is oxidized by tyrosinase to form a highly reactive *ortho*-quinone, (*N*-propionyl-4-*S*- cysteaminylcatechol, NPrCAQ; Figure 8), which then binds covalently to biologically relevant thiols including proteins through the cysteine residues. *In vitro *and* in vivo *studies were also conducted to prove the binding of the quinone-hapten NPrCAQ to proteins. The thiol adducts were analyzed after acid hydrolysis as 5-*S*-cysteaminyl-3-*S*-cysteinylcatechol (CA-CysC) (Figure 8). Our results specifically provided evidence that NPrCAP is oxidized by tyrosinase to an *ortho*-quinone, NPrCAQ, which is highly reactive yet stable enough to survive and then interact with biologically relevant thiols to form covalent adducts. The activation of NPrCAP to NPrCAQ by tyrosinase and the subsequent binding to proteins through cysteine residues were also demonstrated in the* in vitro* and *in vivo* experiments. Our finding was the first demonstration that the quinone-protein adduct formation actually takes place in melanoma cells and melanoma tissues through the tyrosinase-mediated mechanism. Furthermore, 60–80% of the NPrCAQ-thiol adducts were found in the protein fraction in melanoma cells and in the tumors. This is surprising when we consider the much lower reactivity of protein sulfhydryl groups compared with those in small thiols such as cysteine [60, 61]. The remaining nonprotein SH adducts were produced by the reaction of NPrCAQ with free cysteine or glutathione as a detoxifying mechanism. In this connection, it was previously shown that the depletion of glutathione augmented the melanocytotoxicity and antimelanoma effects of NAcCAP [62].

According to the potent melanoma immunotherapy theory using monobenzone [54, 55, 57–59], tyrosinase appears to trigger melanoma regression. Tyrosinase oxidation of monobenzone produces a highly reactive quinone-hapten [44, 54] and ROS concurrently [54]. The quinone-hapten binds to cysteine residues in tyrosinase or other melanosomal proteins thereby generating possible neoantigen, which activate hapten-reactive CD8^+^ T-cells. The latter cells kill monobenzone-exposed melanocytes expressing haptenated antigens on their surface, further liberating melanocyte antigens for presentation by dendritic cells. Finally, the antigen-specific T-cell response is induced and propagated [54, 57–59]. The ROS generated also causes damage to melanosomes leading to the presentation of melanosome-derived antigens and the induction of antigen-specific T-cell responses [58]. 

These immunological events can also be expected to occur for our NPrCAP because the involvement of CD8^+^ T cells and the production of ROS in NPrCAP-treated melanoma cells were demonstrated in our previous study [46]. We expect the production of NPrCAC through redox exchange in melanoma cells and the subsequent production of ROS from the catechol because the closely related catechol, 4-*S*-cysteaminylcatechol, was shown to produce superoxide radicals (which are rapidly converted to hydrogen peroxide) [63]. The thiol adduct RS-NPrCAC, as a catechol, may also contribute to the production of ROS.

## 6. Summary and Conclusion 

Several clinical trials using melanoma peptides or an antibody that blocks cytotoxic T-lymphocyte-associated antigen on lymphocytes have been shown to improve overall melanoma survival [64–66]. Promising oncogene-targeted melanoma therapy has also been successfully introduced recently [67]. 

**Figure 9:**
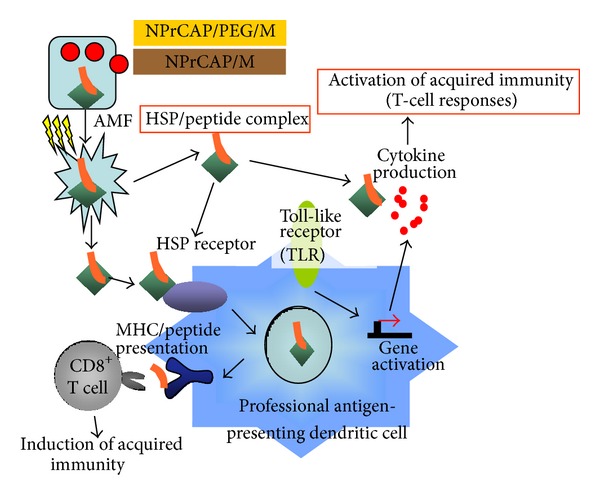
Scheme of intracellular hyperthermia using NPrCAP/PEG/M or NPrCAP/M with AMF exposure. NPrCAP/PEG/M nanoparticles are selectively incorporated in melanoma cells. Intracellular hyperthermia can induce necrotic cell death, and adjacent live melanoma cells suffer heat shock, resulting in increased level of intracellular HSP-peptide complexes. Repeated hyperthermia turns heat-shocked cells to necrotic cells, leading to the release of HSP-peptide complexes into extracellular milieu. The released HSPs-peptide complexes are taken up by dendritic cells (DCs). Then, DCs migrate into regional lymph nodes and cross-present HSP chaperoned antigenic peptides to CD8^+^ T cells in the context of MHC class I molecules, thereby inducing antimelanoma cytotoxic CD8^+^ T cells.

Our study may however indicate that exploitation of a specific biological property to cancer cells can be another approach for developing novel melanoma-targeted drugs which can also trigger the production of melanoma-targeted *in situ* vaccine. Our approach using melanogenesis substrate and magnetite nanoparticles is based upon the expectation of (i) direct killing of melanoma cells by chemotherapeutic and thermo-therapeutic effect of melanogenesis-targeted drug (NPrCAP/M) and (ii) indirect killing by immune reaction (*in situ *peptide vaccine) after exposure to AMF. It is hoped from these rationales that a tumor-specific DDS is developed by NPrCAP, and selective cell death can be achieved by exposure of conjugates of NPrCAP/M nanoparticles to AMF. Hyperthermia increases the expression of intracellular HSPs which is important in and necessary for the induction of antitumor immunity [41, 68]. Overexpression of HSPs increases tumor immunogenicity by augmenting the chaperoning ability of antigenic peptides and presentation of antigenic peptides in MHC class I molecules [39, 69]. In this process professional antigen-presenting dendritic cells play unique and important roles in taking up, processing, and presenting exogenous antigens in association with MHC class I molecules. Our study indicated that combination of melanogenesis substrate, NPrCAP, and local magnetite nanoparticles with hyperthermia could induce* in situ* a form of vaccine against tumor cells and may be effective not only for primary melanoma but also for distant secondary metastases (Figure 9).

Interestingly we found that NPrCAP by itself has potent chemotherapeutic and immune-adjuvant effects. It was demonstrated that the phenol NPrCAP, as a prohapten, can be activated in melanoma cells by tyrosinase to the reactive quinone-hapten NPrCAQ which binds to melanosomal proteins through their cysteine residues to form possible neo-antigens, thus triggering the immunological response (Figure 8).

## Abbreviations

AMF: Alternating magnetic field
BSA: Bovine serum albumin
BSO: Buthionine sulfoximide
CA-CysC: 5-*S*-cysteaminyl-3-*S*-cysteinylcatechol
CAP: Cysteaminylphenol
CDR3: Third complementarity determining region
CML: Cationic magneto-liposome
CTI therapy: Chemothermoimmunotherapy
CTL: Cytotoxic T lymphocyte
DCs: Dendritic cells
DDS: Drug delivery system
DLNs: Draining lymph nodes
HSP/Hsp: Heat shock protein
IL: Interleukin
M: Magnetite nanoparticle
mAb: Monoclonal antibody
MART-1: Melanoma antigen recognized by T cells-1
MCLs: Magnetite cationic liposomes
MC1R: Melanocortin 1 receptor
MHC: Major histocompatibility complex
MITF: Microphthalmia transcription factor
ML: Noncationic magnetoliposome
MSH: Melanocyte stimulating hormone
NAcCAP: N-acetyl-4-S cysteaminylphenol
NDLN: Nondraining lymph node
NPrCAC: N-propionyl-4-S cysteaminylcatechol
NPrCAP: N-propionyl-4-S cysteaminylphenol
NPrCAQ: N-propionyl-4-S-cysteaminylquinine
Nrf2: NF-E2-related factor 2
OVA: Ovu-albumin
PEG: Polyethylene glycol
ROS: Reactive oxygen species
RT-PCR: Reverse transcription polymerase chain reaction
TILs: Tumor-infiltrating lymphocytes
TCRs: T-cell receptors
TRP-2: Tyrosinase-related protein-2.

## References

[1] de Vries E, van de Poll-Franse LV, Louwman WJ, de Gruijl FR, Coebergh JWW (2005). Predictions of skin cancer incidence in the Netherlands up to 2015. *The British Journal of Dermatology*.

[2] Balch CM, Gershenwald JE, Soong SJ (2009). Final version of 2009 AJCC melanoma staging and classification. *Journal of Clinical Oncology*.

[3] Balch CM, Buzaid AC, Soong SJ (2001). Final version of the American joint committee on cancer staging system for cutaneous melanoma. *Journal of Clinical Oncology*.

[4] Reszka K, Jimbow K, Fuchs J, Packer L (1993). Electron donor and acceptor properties of melanin pigments in the skin. *Oxidative Stress in Dermatology*.

[5] Jimbow K, Iwashina T, Alena F, Yamada K, Pankovich J, Umemura T (1993). Exploitation of pigment biosynthesis pathway as a selective chemotherapeutic approach for malignant melanoma. *Journal of Investigative Dermatology*.

[6] Alena F, Iwashina T, Gili A, Jimbow K (1994). Selective *in vivo* accumulation of N-acetyl-4-S-cysteaminylphenol in B16F10 murine melanoma and enhancement of its *in vitro* and *in vivo* antimelanoma effect by combination of buthionine sulfoximine. *Cancer Research*.

[7] Pankovich JM, Jimbow K (1991). Tyrosine transport in a human melanoma cell line as a basis for selective transport of cytotoxic analogues. *Biochemical Journal*.

[8] Tandon M, Thomas PD, Shokravi M (1998). Synthesis and antitumour effect of the melanogenesis-based antimelanoma agent N-Propionyl-4-S-cysteaminylphenol. *Biochemical Pharmacology*.

[9] Gili A, Thomas PD, Ota M, Jimbow K (2000). Comparison of *in vitro* cytotoxicity of N-acetyl and N-propionyl derivatives of phenolic thioether amines in melanoma and neuroblastoma cells and the relationship to tyrosinase and tyrosine hydroxylase enzyme activity. *Melanoma Research*.

[10] Thomas PD, Kishi H, Cao H (1999). Selective incorporation and specific cytocidal effect as the cellular basis for the antimelanoma action of sulphur containing tyrosine analogs. *Journal of Investigative Dermatology*.

[11] Ito A, Shinkai M, Honda H, Kobayashi T (2005). Medical application of functionalized magnetic nanoparticles. *Journal of Bioscience and Bioengineering*.

[12] Kawai N, Ito A, Nakahara Y (2005). Anticancer effect of hyperthermia on prostate cancer mediated by magnetite cationic liposomes and immune-response induction in transplanted syngeneic rats. *Prostate*.

[13] Shinkai M, Yanase M, Honda H, Wakabayashi T, Yoshida J, Kobayashi T (1996). Intracellular hyperthermia for cancer using magnetite cationic liposomes: *in vitro* study. *Japanese Journal of Cancer Research*.

[14] Ménoret A, Chandawarkar R (1998). Heat-shock protein-based anticancer immunotherapy: an idea whose time has come. *Seminars in Oncology*.

[15] Srivastava PK, Ménoret A, Basu S, Binder R, Quade K (1998). Heat shock proteins come of age: primitive functions acquired new roles in an adaptive world. *Immunity*.

[16] Tamura Y, Tsuboi N, Sato N, Kikuchi K (1993). 70 kDa heat shock cognate protein is a transformation-associated antigen and a possible target for the host’s anti-tumor immunity. *Journal of Immunology*.

[17] Tamura Y, Peng P, Liu K, Daou M, Srivastava PK (1997). Immunotherapy of tumors with autologous tumor-derived heat shock protein preparations. *Science*.

[18] Tamura Y, Sato N (2003). Heat shock proteins: chaperoning of innate and adaptive immunities. *Japanese Journal of Hyperthermic Oncology*.

[19] Srivastava PK (2005). Immunotherapy for human cancer using heat shock protein-peptide complexes. *Current Oncology Reports*.

[20] Tamura Y, Takashima S, Cho JM (1996). Inhibition of natural killer cell cytotoxicity by cell growth-related molecules. *Japanese Journal of Cancer Research*.

[21] Ueda G, Tamura Y, Hirai I (2004). Tumor-derived heat shock protein 70-pulsed dendritic cells elicit-tumor-specific cytotoxic T lymphocytes (CTLs) and tumor immunity. *Cancer Science*.

[22] Ito A, Honda H, Kobayashi T (2006). Cancer immunotherapy based on intracellular hyperthermia using magnetite nanoparticles: a novel concept of “heat-controlled necrosis” with heat shock protein expression. *Cancer Immunology, Immunotherapy*.

[23] Shi H, Cao T, Connolly JE (2006). Hyperthermia enhances CTL cross-priming. *Journal of Immunology*.

[24] Dakour J, Vinayagamoorthy T, Jimbow K (1993). Identification of a cDNA coding for a Ca^2+^-binding phosphoprotein (p90) calnexin, on melanosomes in normal and malignant human melanocytes. *Experimental Cell Research*.

[25] Toyofuku K, Wada I, Hirosaki K, Park JS, Hori Y, Jimbow K (1999). Promotion of tyrosinase folding in Cos 7 cells by calnexin. *Journal of Biochemistry*.

[26] Jimbow K, Gomez PF, Toyofuku K (1997). Biological role of tyrosinase related protein and its biosynthesis and transport from TGN to stage I melanosome, late endosome, through gene transfection study. *Pigment Cell Research*.

[27] Jimbow K, Park JS, Kato F (2000). Assembly, target-signaling and intracellular transport of tyrosinase gene family proteins in the initial stage of melanosome biogenesis. *Pigment Cell Research*.

[28] Jimbow K, Hua C, Gomez PF (2000). Intracellular vesicular trafficking of tyrosinase gene family protein in Eu- and pheomelanosome biogenesis. *Pigment Cell Research*.

[29] Miura T, Jimbow K, Ito S (1990). The *in vivo* antimelanoma effect of 4-S-cysteaminylphenol and its N-acetyl derivative. *International Journal of Cancer*.

[30] Ito S, Kato T, Ishikawa K, Kasuga T, Jimbow K (1987). Mechanism of selective toxicity of 4-S-cysteinylphenol and 4-S-cysteaminylphenol to melanocytes. *Biochemical Pharmacology*.

[31] Minamitsuji Y, Toyofuku K, Sugiyama S, Yamada K, Jimbow K (1999). Sulfur containing tyrosine analogs can cause selective melanocytotoxicity involving tyrosinase-mediated apoptosis. *Journal of Investigative Dermatology Symposium Proceedings*.

[32] Jimbow K, Miyake Y, Homma K (1984). Characterization of melanogenesis and morphogenesis of melanosomes by physicochemical properties of melanin and melanosomes in malignant melanoma. *Cancer Research*.

[33] Thiesen B, Jordan A (2008). Clinical applications of magnetic nanoparticles for hyperthermia. *International Journal of Hyperthermia*.

[34] Johannsen M, Gneveckow U, Eckelt L (2005). Clinical hyperthermia of prostate cancer using magnetic nanoparticles: presentation of a new interstitial technique. *International Journal of Hyperthermia*.

[35] Ito A, Fujioka M, Yoshida T (2007). 4-S-Cysteaminylphenol-loaded magnetite cationic liposomes for combination therapy of hyperthermia with chemotherapy against malignant melanoma. *Cancer Science*.

[36] Sato M, Yamashita T, Ohkura M (2009). N-propionyl-cysteaminylphenol-magnetite conjugate (NPrCAP/M) Is a nanoparticle for the targeted growth suppression of melanoma cells. *Journal of Investigative Dermatology*.

[37] Takada T, Yamashita T, Sato M (2009). Growth inhibition of re-challenge B16 melanoma transplant by conjugates of melanogenesis substrate and magnetite nanoparticles as the basis for developing melanoma-targeted chemo-thermo-immunotherapy. *Journal of Biomedicine and Biotechnology*.

[38] Suzuki M, Shinkai M, Honda H, Kobayashi T (2003). Anticancer effect and immune induction by hyperthermia of malignant melanoma using magnetite cationic liposomes. *Melanoma Research*.

[39] Yanase M, Shinkai M, Honda H, Wakabayashi T, Yoshida J, Kobayashi T (1998). Intracellular hyperthermia for cancer using magnetite cationic liposomes: an *in vivo* study. *Japanese Journal of Cancer Research*.

[40] Hergt R, Dutz S, Müller R, Zeisberger M (2006). Magnetic particle hyperthermia: nanoparticle magnetism and materials development for cancer therapy. *Journal of Physics Condensed Matter*.

[41] Ito A, Shinkai M, Honda H (2003). Heat shock protein 70 expression induces antitumor immunity during intracellular hyperthermia using magnetite nanoparticles. *Cancer Immunology, Immunotherapy*.

[42] Ito A, Matsuoka F, Honda H, Kobayashi T (2004). Antitumor effects of combined therapy of recombinant heat shock protein 70 and hyperthermia using magnetic nanoparticles in an experimental subcutaneous murine melanoma. *Cancer Immunology, Immunotherapy*.

[43] Sato A, Tamura Y, Sato N (2010). Melanoma-targeted chemo-thermo-immuno (CTI)-therapy using N-propionyl-4-S-cysteaminylphenol-magnetite nanoparticles elicits CTL response via heat shock protein-peptide complex release. *Cancer Science*.

[44] Manini P, Napolitano A, Westerhof W, Riley PA, D’Ischia M (2009). A reactive ortho-quinone generated by tyrosinase-catalyzed oxidation of the skin depigmenting agent monobenzone: self-coupling and thiol-conjugation reactions and possible implications for melanocyte toxicity. *Chemical Research in Toxicology*.

[45] Ito A, Yamaguchi M, Okamoto N (2012). T-cell receptor repertoires of tumor-infiltrating lymphocytes after hyperthermia using functionalized magnetite nanoparticles. *Nanomedicine*.

[46] Ishii-Osai Y, Yamashita T, Tamura Y (2012). N-Propionyl-4-S-cysteaminylphenol induces apoptosis in B16F1 cells and mediates tumor-specific T-cell immune responses in a mouse melanoma model. *Journal of Dermatological Science*.

[47] Ito S, Nishigaki A, Ishii-Osai Y (2012). Mechanism of putative neo-antigen formation from N-propionyl-4-S-cysteaminylphenol, a tyrosinase substrate, in melanoma models. *Biochemical Pharmacology*.

[48] Jimbow K, Obata H, Pathak MA, Fitzpatrick TB (1974). Mechanism of depigmentation by hydroquinone. *Journal of Investigative Dermatology*.

[49] Nordlund JJ, Forget B, Kirkwood J, Lerner AB (1985). Dermatitis produced by applications of monobenzone in patients with active vitiligo. *Archives of Dermatology*.

[50] Boissy RE, Manga P (2004). On the etiology of contact/occupational vitiligo. *Pigment Cell Research*.

[51] Cooksey CJ, Jimbow K, Land EJ, Riley PA (1993). Reactivity of orthoquinones involved in tyrosinase-dependent cytotoxicity: differences between alkylthio- and alkoxy-substituents. *Melanoma Research*.

[52] Parsons PG, Favier D, McEwan M, Takahashi H, Jimbow K, Ito S (1991). Action of cysteaminylphenols on human melanoma cells *in vivo* and *in vitro*: 4-S-cysteaminylphenol binds protein disulphide isomerase. *Melanoma research*.

[53] Hariharan V, Klarquist J, Reust MJ (2010). Monobenzyl ether of hydroquinone and 4-tertiary butyl phenol activate markedly different physiological responses in melanocytes: relevance to skin depigmentation. *Journal of Investigative Dermatology*.

[54] van den Boorn JG, Picavet DI, van Swieten PF (2011). Skin-depigmenting agent monobenzone induces potent T-cell autoimmunity toward pigmented cells by tyrosinase haptenation and melanosome autophagy. *Journal of Investigative Dermatology*.

[55] van den Boorn JG, Konijnenberg D, Tjin EP (2010). Effective melanoma immunotherapy in mice by the skin-depigmenting agent monobenzone and the adjuvants imiquimod and CpG. *PloS ONE*.

[56] Quaglino P, Marenco F, Osella-Abate S (2010). Vitiligo is an independent favourable prognostic factor in stage III and IV metastatic melanoma patients: results from a single-institution hospital-based observational cohort study. *Annals of Oncology*.

[57] Westerhof W, Manini P, Napolitano A, d’Ischia M (2011). The haptenation theory of vitiligo and melanoma rejection: a close-up. *Experimental Dermatology*.

[58] van den Boorn JG, Melief CJ, Luiten RM (2011). Monobenzone-induced depigmentation: from enzymatic blockage to autoimmunity. *Pigment Cell and Melanoma Research*.

[59] Westerhof W, D’Ischia M (2007). Vitiligo puzzle: the pieces fall in place. *Pigment Cell Research*.

[60] Kato T, Ito S, Fujita K (1986). Tyrosinase-catalyzed binding of 3,4-dihydroxyphenylalanine with proteins through the sulfhydryl group. *Biochimica et Biophysica Acta*.

[61] Ito S, Kato T, Fujita K (1988). Covalent binding of catechols to proteins through the sulphydryl group. *Biochemical Pharmacology*.

[62] Alena F, Jimbow K, Ito S (1990). Melanocytotoxicity and antimelanoma effects of phenolic amine compounds in mice *in vivo*. *Cancer Research*.

[63] Hasegawa K, Ito S, Inoue S, Wakamatsu K, Ozeki H, Ishiguro I (1997). Dihydro-1,4-benzothiazine-6,7-dione, the ultimate toxic metabolite of 4-S-cysteaminylphenol and 4-S-cysteaminylcatechol. *Biochemical Pharmacology*.

[64] Hodi FS, O’Day SJ, McDermott DF (2010). Improved survival with ipilimumab in patients with metastatic melanoma. *The New England Journal of Medicine*.

[65] Schwartzentruber DJ, Lawson DH, Richards JM (2011). gp100 peptide vaccine and interleukin-2 in patients with advanced melanoma. *The New England Journal of Medicine*.

[66] Robert C, Thomas L, Bondarenko I (2011). Ipilimumab plus dacarbazine for previously untreated metastatic melanoma. *The New England Journal of Medicine*.

[67] Chapman PB, Hauschild A, Robert C (2011). Improved survival with vemurafenib in melanoma with BRAF V600E mutation. *The New England Journal of Medicine*.

[68] Mise K, Kan N, Okino T (1990). Effect of heat treatment on tumor cells and antitumor effector cells. *Cancer Research*.

[69] Ito A, Matsuoka F, Honda H, Kobayashi T (2003). Heat shock protein 70 gene therapy combined with hyperthermia using magnetic nanoparticles. *Cancer Gene Therapy*.

